# Stromal composition predicts recurrence of early rectal cancer after local excision

**DOI:** 10.1111/his.14438

**Published:** 2021-09-03

**Authors:** Helen J S Jones, Chris Cunningham, Hanne A Askautrud, Håvard E Danielsen, David J Kerr, Enric Domingo, Tim Maughan, Simon J Leedham, Viktor H Koelzer

**Affiliations:** ^1^ Department of Colorectal Surgery Oxford University Hospitals NHS Trust Oxford UK; ^2^ Institute for Cancer Genetics and Informatics Oslo University Hospital Oslo Norway; ^3^ Department of Informatics University of Oslo Oslo Norway; ^4^ Nuffield Division of Clinical Laboratory Sciences University of Oxford Oxford UK; ^5^ Department of Oncology MRC Oxford Institute for Radiation Oncology University of Oxford Oxford UK; ^6^ Intestinal Stem Cell Biology Laboratory Nuffield Department of Medicine Wellcome Centre for Human Genetics University of Oxford Oxford UK; ^7^ Department of Pathology and Molecular Pathology University and University Hospital Zürich Zürich Switzerland; ^8^ Department of Oncology and Nuffield Department of Medicine University of Oxford Oxford UK

**Keywords:** desmoplastic stroma, early rectal cancer, inflamed stroma local excision, prognostic factor, stroma

## Abstract

**Aims:**

After local excision of early rectal cancer, definitive lymph node status is not available. An alternative means for accurate assessment of recurrence risk is required to determine the most appropriate subsequent management. Currently used measures are suboptimal. We assess three measures of tumour stromal content to determine their predictive value after local excision in a well‐characterised cohort of rectal cancer patients without prior radiotherapy.

**Methods and results:**

A total of 143 patients were included. Haematoxylin and eosin (H&E) sections were scanned for (i) deep neural network (DNN, a machine‐learning algorithm) tumour segmentation into compartments including desmoplastic stroma and inflamed stroma; and (ii) digital assessment of tumour stromal fraction (TSR) and optical DNA ploidy analysis. 3′ mRNA sequencing was performed to obtain gene expression data from which stromal and immune scores were calculated using the ESTIMATE method. Full results were available for 139 samples and compared with disease‐free survival. All three methods were prognostic. Most strongly predictive was a DNN‐determined ratio of desmoplastic to inflamed stroma >5.41 (*P* < 0.0001). A ratio of ESTIMATE stromal to immune score <1.19 was also predictive of disease‐free survival (*P* = 0.00051), as was stromal fraction >36.5% (*P* = 0.037).

**Conclusions:**

The DNN‐determined ratio of desmoplastic to inflamed ratio is a novel and powerful predictor of disease recurrence in locally excised early rectal cancer. It can be assessed on a single H&E section, so could be applied in routine clinical practice to improve the prognostic information available to patients and clinicians to inform the decision concerning further management.

## Graphical abstract

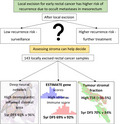

## Introduction

Local excision (LE) for early rectal cancer (ERC) is gaining in popularity as patients and clinicians seek to avoid traditional radical surgery with its morbidity. However, by not removing the mesorectum the definitive nodal status remains unknown, and there is a higher risk of local recurrence due to occult metastases.^1^ This may be mitigated by close surveillance to detect any recurrence early when amenable to salvage surgery, or more aggressively by adjuvant chemoradiotherapy (CRT) or completion radical surgery. These latter options carry some morbidity, so prognostic features more accurate than are currently used^2^ are sought to more effectively guide their use.

In many types of solid cancer, a higher proportion of stroma correlates with poorer prognosis.^3^ The usual measure of stromal contribution is tumour stromal ratio (TSR); this can be obtained from haematoxylin and eosin (H&E)‐stained slides. It has been proposed that TSR should be included in the TNM (tumour–node–metastasis) staging algorithm to improve prognostic information.^4^ One drawback is low interobserver agreement between pathologists.^5^ Promising digital methods have been developed; in a study of more than 1800 colorectal cancers, tumours with more than 65% stroma had almost double the risk of recurrence (42% versus 22%) in 10 years compared with those with <50% stroma.^6^ In early colorectal cancer, a combined measure of TSR and epithelial ploidy may be more effective than TSR alone.^7^

An artificial intelligence‐based method of tissue segmentation within the tumour has been developed and shown to accurately measure tissue compartments in colorectal cancer.^8^, ^9^ This segmentation includes two types of stroma: desmoplastic and inflamed. Desmoplastic stroma is characterised by disorganised production of connective tissue, comprising mainly collagen fibres, and has been assessed in colorectal cancer.^10^ The main cells are cancer‐associated fibroblasts (CAF).^11^ Inflamed stroma is characterised by a lymphocyte‐rich infiltrate.

Gene expression has also been used to assess the stroma and immune components of the tumour. The ESTIMATE stromal and immune scores use expression data for 141 genes each to infer the fraction of stromal and immune cells in a tumour^12^ and have shown prognostic utility in colorectal cancer.^13^

Previous studies have assessed colorectal cancer in general, and it is not clear whether these results are applicable to patients with ERC suitable for organ preserving surgery. After LE, currently used prognostic features stratify patients’ recurrence risk and are used to advise on further management, but these are less than adequate: some patients undergo radical completion surgery to find no residual tumour,^14^ while others undergo surveillance for low‐risk disease yet develop recurrence.^15^ The aim of this study is to assess the value of stromal content in locally excised ERC as a biomarker for recurrence risk. An accurate and clinically applicable biomarker would be a valuable adjunct to inform the decision‐making process regarding subsequent management after LE.

## Materials and methods

### ETHICS

This study was approved by West Midlands–South Birmingham Research Ethics Committee as: ‘An observational study to correlate the results of ploidy and stroma analysis with prognosis in early rectal cancer’ (16/WM/0443, 28/10/2016) and ‘Pre‐treatment molecular stratification and the histogenic origins of rectal cancer’ as an umbrella project approved by Oxford University Research tissue bank ethics reference 11/YH/0020 and IBD Cohort 09/H1204/30.

### PATIENT COHORT

The Oxford transanal endoscopic microsurgery (TEM) database prospectively collects data on all patients undergoing LE for rectal cancer. All patients were considered to have early rectal cancer suitable for LE based on pre‐operative imaging. Data include demographics, operative details, histopathological data and follow‐up. All those who had surgery between 2007 and 2017 and consented to tissue use for ethically approved research were eligible; any patient who had prior radiotherapy was excluded. Formalin‐fixed paraffin‐embedded tissue blocks containing tumour were retrieved. Sequential sections were cut; two 5‐μm sections were stained with H&E and further sections used for RNA extraction.

### DNN DIGITAL PATHOLOGY

**Figure 1:**
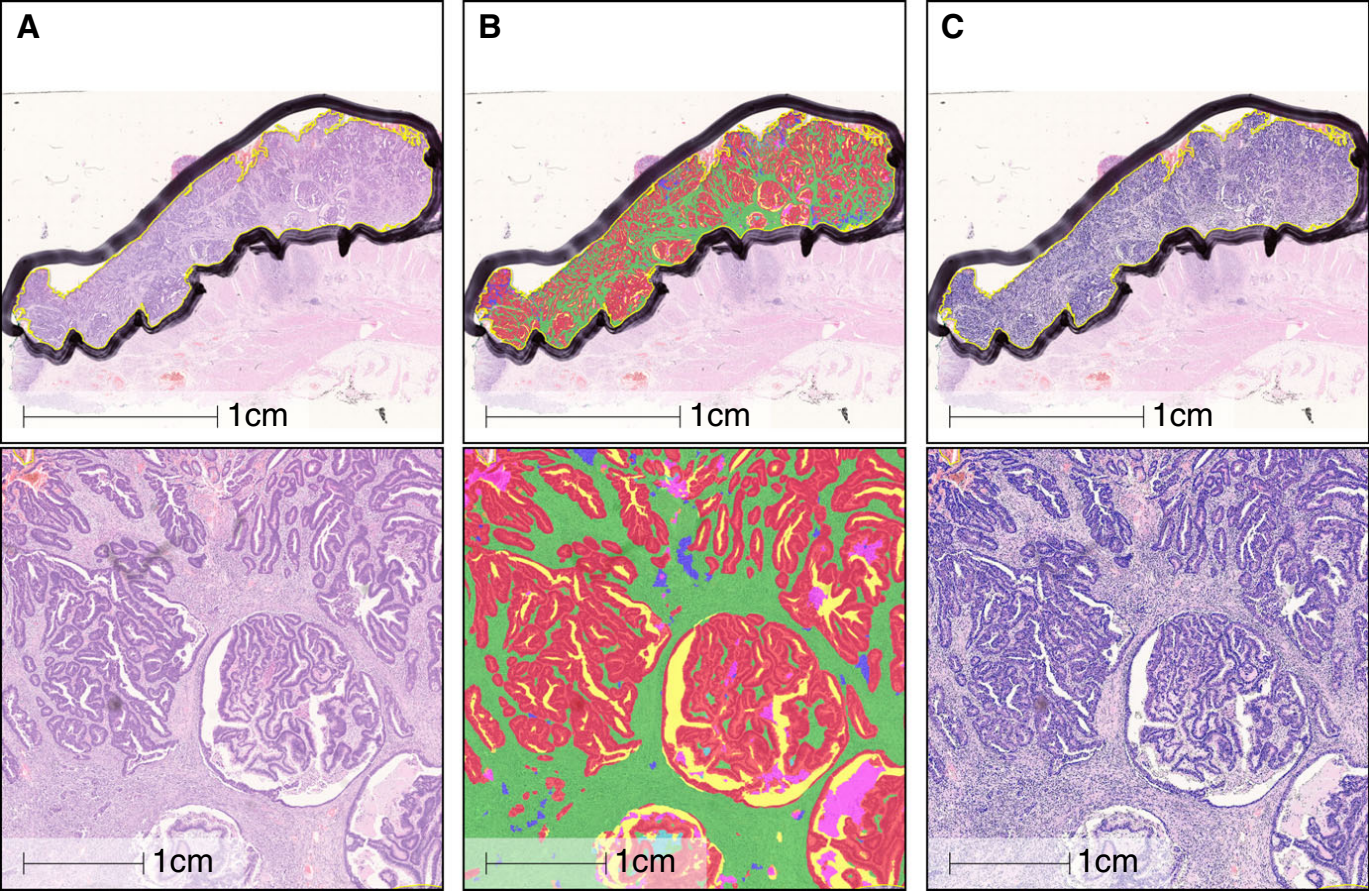
Example of a tumour annotated using a deep neural net algorithm, showing overview and zoomed view. **A**, Unannotated original; **B**, deep neural network (DNN) annotation; **C**, cell segmentation. Tissue compartments: red = epithelium; green = desmoplastic stroma; purple = inflamed stroma; blue = mucin; yellow = white space; pink = necrosis.

A stained section was annotated to indicate the cancer then scanned. Artificial intelligence (AI)‐based histomorphological tissue classification was undertaken using a deep neural net algorithm (DNN)^8^ to quantify tissue composition across the whole lesion. DNN automatically segments the tumour into the following compartments (Figure 1) and quantifies the area (mm^2^) with a maximum resolution of 50 µm^2^ for a single area:

1. Background (white space, excluded from subsequent analysis).
2. Necrosis.
3. Epithelium (tumour area).
4. Desmoplastic stroma.
5. Inflamed stroma.
6. Mucin.
7. Non‐neoplastic mesenchymal components of bowel wall.

### Stromal Fraction (TSR)

**Figure 2:**
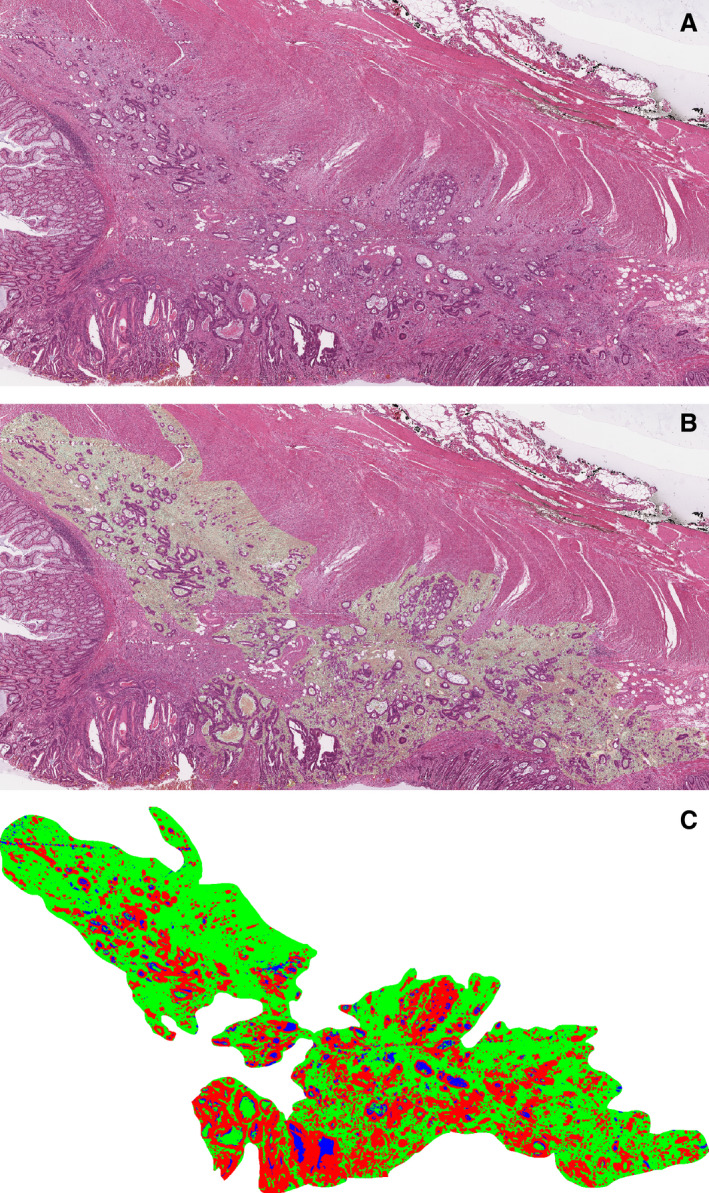
Example of a tumour with a high stromal fraction (60%) by tumour stromal ratio (TSR). **A**, Scan of haematoxylin and eosin (H&E) slide from the tumour; **B**, digitally processed scan showing segmented stroma in light green; **C**, mask of tumour produced by digital processing, with stroma in green. Red indicates tumour cells and blue is artefact. The mask was used to calculate the tumour stromal fraction.

A stained section was scanned and digitally annotated. Two masks were created for background and connective tissue. After further image processing the masks were combined and TSR calculated according to previously published methodology^7^ (Figure 2).

### PLOIDY

A section was used for optical density ploidy analysis according to previously published methodology^16^ and classification criteria.^17^ The DNA ploidy histogram for each sample was classified as diploid or non‐diploid. The ploidy status was combined with the TSR, using a 50% stromal fraction cut‐off into four groups with the intermediate two combined: diploid low stroma, diploid high stroma + non‐diploid low stroma, non‐diploid high stroma.^7^

### GENE EXPRESSION

Five slides were deparaffinised then dissected using a 21‐gauge needle and the marked H&E slide as a guide to extract tumour tissue. RNA was extracted using Roche High Pure FFPET RNA isolation kit (version 3, October 2012, modified protocol; Roche, Basel, Switzerland). The extract was treated with Invitrogen Amplification Grade DNase treatment (ThermoFisher, Fremont, CA, USA; catalogue number: 18068015) to remove DNA. The extracted RNA was submitted for 3′ mRNA sequencing, using reference genome GRCh37.EBVB95‐8wt.ERCC.

### SIGNATURE SCORES

ESTIMATE scores^12^ for stromal and immune signature were calculated using the ESTIMATE R package.^18^ Each signature is based on 141 genes. The sum of the stromal and immune scores is used to infer tumour purity.

### STATISTICAL ANALYSIS

Most results were available for most patients, but for a few, one or more of the results were unavailable; data are given for all available patients for each technique. Data were collated and descriptive statistics obtained in Excel (Microsoft). Summary data are reported as median and interquartile range (IQR). Analysis was undertaken using R statistical software (www.r‐project.org). The Mann–Whitney test was used to test for group differences. Receiving operating characteristic (ROC) curves were used to assess the area under the curve (AUC) and optimal cut‐points using the Youden index. Cox proportional‐hazards regression was used to assess outcome and Kaplan–Meier survival estimates were obtained. Akaike’s information criterion (AIC) was used to compare the fit of survival models. Univariable analysis assessed association with disease recurrence. Disease‐free survival (DFS) was calculated from date of LE to date of detection of local or distant recurrence, or censoring. Patients without recurrence were censored at the date of last follow‐up or death.

## RESULTS

Suitable TEM tissue samples were available from 150 patients. TSR results were available for 143 and ploidy for 140 of these. DNN analysis results were available for 140; there was an overlap of 139 between these groups. ESTIMATE stroma and immune scores were available for all patients. Table 1 shows demographic, tumour and outcome data.

**Table 1:** Demographic, tumour and outcome data for the patient group

	Number (%)
Number of patients	143
Age, median (IQR)	69 (64–78)
Sex male (%):female	87 (61%): 56
Tumour height above anal verge	
<6.0 cm	68 (48%)
>6 cm	75 (52%)
Tumour stage	
pT1	76 (53%)
pT1 sm1	11 (8%)
pT1 sm2	21 (15%)
pT1 sm3	44 (31%)
pT2	58 (41%)
pT3	9 (6%)
Tumour diameter (cm)	
<3.0	113 (79%)
3–5	26 (18%)
>5	4 (3%)
Histological subtype^23^	
Adenocarcinoma NOS	113 (79%)
Mucinous adenocarcinoma	1 (1%)
Adenocarcinoma with mucinous component	13 (9%)
Serrated adenocarcinoma	12 (8%)
Adenoma‐like adenocarcinoma	4 (3%)
Differentiation	
Well‐differentiated	12 (9%)
Moderately differentiated	123 (86%)
Poorly differentiated	8 (8%)
Lymphovascular invasion	44 (31%)
Resection margin <1 mm	41 (29%)
Post‐TEM management	
Completion surgery	13 (9%)
Adjuvant radiotherapy	42 (29%)
Surveillance alone	88 (62%)
Local recurrence	18 (13%)
Distant metastases	14 (10%)
Length of follow‐up, years, median (IQR)	4.0 (2.2–5.3)

IQR, Interquartile range; NOS, Not otherwise specified; TEM, Transanal endoscopic microsurgery.

### DNN ANALYSIS

**Figure 3:**
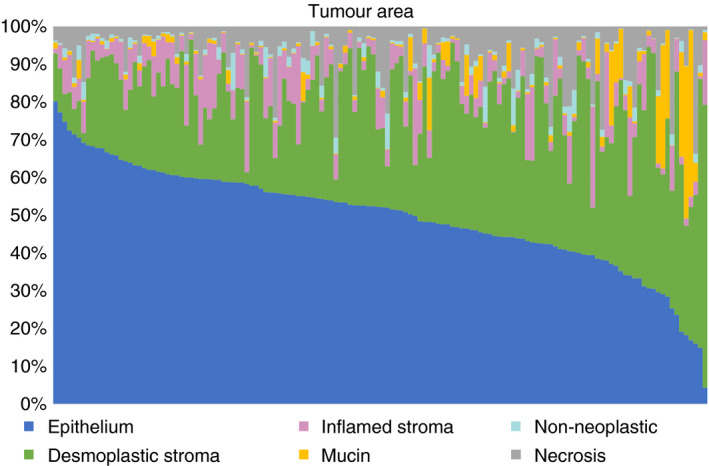
Proportional area of the tumour components determined by deep neural network (DNN) digital pathology in 140 rectal cancers. In most tumours, epithelium forms the major part and epithelial cells predominate. Desmoplastic stroma forms a significant part of most tumours while inflamed stroma makes up a smaller, variable proportion. Few tumours are mucinous. Non‐neoplastic tissue and necrosis form only a minor part in most annotated tumour regions. [Colour figure can be viewed at wileyonlinelibrary.com]

The proportional classified areas are shown in Figure 3. The median proportion of epithelium was 0.52 (IQR = 0.42–0.59). Considering only the cellular areas, the median epithelial tumour content was 56% (IQR = 49–64%). For desmoplastic stroma, the median proportional area was 0.30 (IQR = 0.22–0.40) and for inflammatory stroma was 0.05 (IQR = 0.02–0.09).

**Figure 4:**
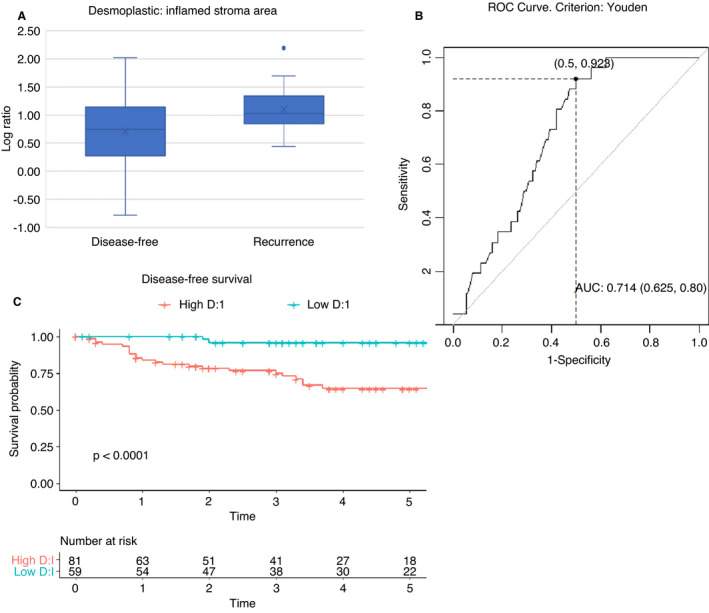
Log ratio of desmoplastic to inflamed stroma area in rectal cancer. **A**, Box and whisker plot showing log ratio of desmoplastic to inflamed stroma determined by deep neural network (DNN) (D:I) for stromal area in 140 rectal cancers. The median log D:I was 1.01 in 26 patients who developed recurrence and 0.74 in 114 who remained disease‐free. The log D:I was significantly higher in the recurrence group, *P* = 0.00067. **B**, Receiver operating characteristic (ROC) curve assessing log D:I as predictor of recurrence. Using the Youden criterion, the optimal cut‐off is log D:I = 0.73, with area under the curve (AUC) = 0.71, **C**, disease‐free survival by D:I, with high D:I defined as ≥5.41, the cut‐off value on the ROC curve. [Colour figure can be viewed at wileyonlinelibrary.com]

The ratio of desmoplastic to inflamed (D:I) stromal area was calculated and compared with the occurrence of disease recurrence. Figure 4A shows the log of the ratio for the two groups; there was significantly more desmoplastic stroma relative to inflamed in those with recurrence (*P* = 0.00067).

Assessing the value of this ratio as a predictor of recurrence produced the ROC curve shown in Figure 4B. This determines a D:I cut‐off at 5.41 (0.73 for log ratio). AUC is 0.71 [95% confidence interval (CI) = 0.63–0.80]. At this cut‐off, the sensitivity is 0.92, specificity 0.50, positive predictive value (PPV) 0.30 and negative predictive value (NPV) 0.97. This model defined a poor prognosis group, with high D:I comprising 58% (81 of 140) of the total. The DFS curve shows significant survival advantage in those with a low ratio (Figure 4C). The 5‐year DFS for the high D:I group is 65% (95% CI = 54–78) compared with 96% (95% CI = 90–100) for the low D:I group. The AIC for this model was 221. The analysis was repeated using cell count rather than area, and similar results obtained.

### STROMA FRACTION (TSR)

**Figure 5:**
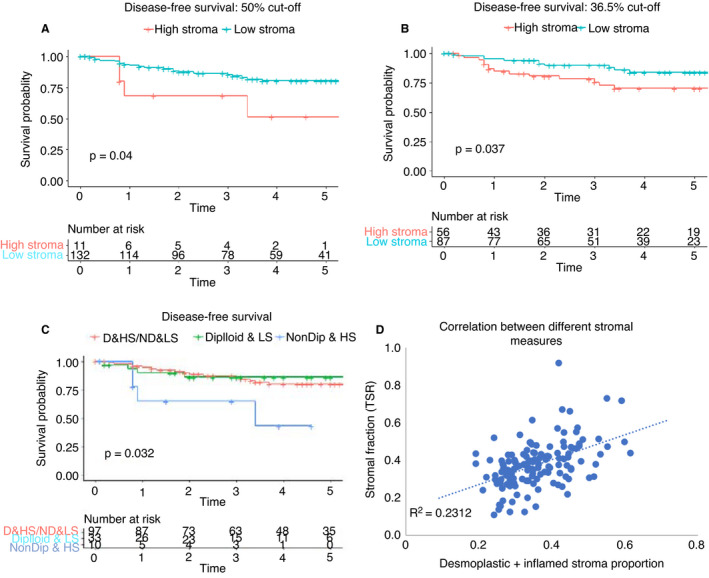
Tumour stromal fraction in rectal cancer. **A**, Disease‐free survival by stromal fraction using a cut‐off of 50% stromal fraction; **B**, Disease‐free survival by stromal fraction using a cut‐off of 36.5% stromal fraction; **C**, disease‐free survival by stromal fraction and ploidy; D = diploid; ND = non‐diploid; LS = low stroma; HS = high stroma. **D**, Scatter‐plot showing correlation of DNN stroma (desmoplastic + inflamed) proportion with tumour stromal fraction. [Colour figure can be viewed at wileyonlinelibrary.com]

TSR for this group varied from 19% to 62% stroma, with a median of 35%. Using the conventional 50% cut‐off, 11 (7.7%) tumours were classified as high stroma and had worse DFS (Figure 5A). These comprised three T1, five T2 and three T3 tumours. ROC suggested an alternate cut‐off at 36.5%; this selected 56 (39%) into the high‐risk group (Figure 5B). The estimated 5‐year DFS in the 50% cut‐off high‐risk group was 51% (95% CI = 25–100) compared with 81% (95% CI = 73–89) for low stromal tumours; model AIC = 239. Corresponding figures using a 36.5% cut‐off were 70% (95% CI = 58–85) 5‐year DFS for the high‐risk group compared with 84% (95% CI = 75–93) for low stromal tumours; AIC was 238.

As previous work^7^ has suggested that in early‐stage cancer a combination of ploidy and TSF may provide better prognostic value than TSR alone this was also assessed, categorising samples as high and low stroma (50% cut‐off) and diploid or non‐diploid. Figure 5C shows a significant difference in DFS, but again with a small high‐risk group (10 of 140, 7%); AIC = 229. Using the 36.5% cut‐off yielded a higher proportion in the non‐diploid, high stroma group (42 of 140, 30%), but did not show a significant difference in DFS (*P* = 0.16).

The proportional tumour area of stroma (desmoplastic and inflamed) obtained via DNN methodology was compared with the stromal fraction. Figure 5D shows a weak positive correlation (*R* ^2^ = 0.23).

### ESTIMATE SCORING FOR TUMOUR COMPARTMENTS

**Figure 6:**
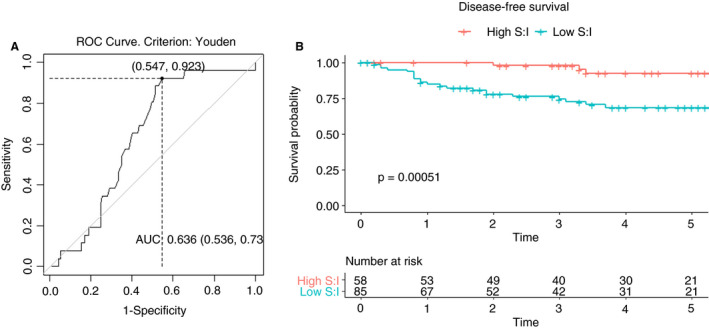
Ratio of ESTIMATE stroma and immune scores in rectal cancers. **A**, Receiver operating characteristic (ROC) curve assessing ratio of ESTIMATE stromal to immune scores (S:I) as predictor of recurrence. Using the Youden criterion, the optimal cut‐off is S:I = 1.24, with area under the curve (AUC) = 0.64. **B**, Disease‐free survival by S:I, with low S:I defined as ≥1.24, the cut‐off value on the ROC curve. [Colour figure can be viewed at wileyonlinelibrary.com]

The median stromal score was −465 (IQR = −994 to 539) and immune score −432 (IQR = −817 to 251). These scores were compared with the occurrence of disease recurrence; no statistically significant association for either score was observed. However, the S:I (stroma:immune) ratio showed a significant association with recurrence (*P* = 0.03) with median S:I = 1.15 in the disease‐free group and 0.96 in the recurrence group. ROC curve (Figure 6A) showed AUC = 0.64 and indicated an optimal cut‐off of 1.24. This classified 85 (59%) tumours as low S:I (high risk); this group had an estimated 5‐year DFS of 69% (95% CI = 58–81) compared with 92% (95% CI = 85–100) for the high S:I group. The sensitivity was 0.92, specificity 0.45, PPV 0.27 and NPV 0.96. DFS curves are shown in Figure 6B, AIC = 228.

The ESTIMATE stromal and immune scores were compared with DNN cell count proportion in desmoplastic and inflamed stroma (Figure S1); these showed a modest positive correlation (*R* ^2^ = 0.37 and 0.21, respectively). A comparison with the proportional area of the stromal compartments showed weaker correlations than the cell count proportion. The median tumour purity, calculated from the ESTIMATE scores, was 0.89 (IQR = 0.87–0.91). This is higher than the DNN‐measured epithelial area and the two measures had a moderate correlation (*R* ^2^ = 0.38).

Table 2 summarises the predictive performance for each of the three stromal measures considered. Table S1 shows the results of univariable analysis for association with disease recurrence and finds only pT stage, positive resection margin and D:I to be significant.

**Table 2:** Summary of prognostic value of three stromal measures in early rectal cancer

Measure	AUC	Proportion at high risk	Estimated 5‐year DFS (95% CI)	AIC	*P*‐value
Stromal fraction >36.5%	0.58	56%	70% (58–85) versus 84% (75–93)	238	0.037
Stroma fraction >50% + ploidy		7%	65% (39–100) versus 86% (79–94) versus 86% (75–100)*	229	0.032
Desmoplastic: inflamed stroma area ratio >5.41	0.71	56%	65% (54–78) versus 96% (90–100)	221	0.00067
ESTIMATE stromal: immune score <1.24	0.64	59%	69% (58–81) versus 92% (85–100)	228	0.00051

AUC, Area under the curve; DFS, Disease‐free survival; CI, Confidence interval; AIC, Akaike information criterion.

* Three‐year survival results shown.

## Discussion

This study has addressed a particular patient group – those undergoing LE for ERC. While all tumours were assessed as suitable for LE on pre‐operative staging, with patients fully informed of the implications, histopathology sometimes shows the tumour to be more advanced or aggressive than expected,^2^ or the resection margin to be less than ideal, especially if the tumour location is low on the sphincter or high, where there is risk of peritoneal breach. Following surgery, patient and clinician must decide how to proceed; whether surveillance alone will suffice or adjuvant treatment should be employed. The present results offer a refinement to the current assessment of recurrence risk based on tumour stage, size and lymphovascular invasion.^19^ The additional consideration of stromal composition may provide a more individualised risk assessment.

In this series of 143 patients, the D:I ratio provided good discrimination between good and poor prognosis tumours with 5‐year estimated DFS of 96 and 65%, respectively. High desmoplastic stromal content is often associated with activated fibroblasts, transforming growth factor (TGF)‐β signalling activation and a poor prognosis.^20^ In contrast, inflamed stroma is rich in lymphocytes; immune activation tends to be associated with a favourable outcome.^21^

TSR provided discrimination between high‐ and low‐risk groups, with 5‐year DFS estimates of 70 and 84%. Tumours with more than 36.5% stroma had poorer prognosis, in keeping with Scheer’s^5^ finding of significantly worse disease‐free and overall survival, with TSR more than 30% among 154 patients with rectal cancer. It is worth noting that only 13% of tumours in our series had a stromal fraction more than 50%, lower than the 34% of 377 rectal cancers in the QUASAR trial,^6^ probably reflecting the earlier cancers in the current series, which may not yet have produced a great volume of stroma. Furthermore, in early cancers more subtle features of the stroma such as the presence of inflammatory infiltrates rather than simply volume may have a greater role. The addition of ploidy to TSR identified a small group (7%) of non‐diploid, high stroma tumours which had poorer outcomes, but this stratification was weaker than for more advanced colorectal cancers.^7^

Although the individual stromal and immune ESTIMATE scores did not show a significant association with survival, a ratio of the two discriminated high‐ and low‐risk groups. The disadvantage of this technique is the requirement for gene expression data which involves time and cost, making it less useful for current clinical practice. The ESTIMATE scores can be used to infer tumour purity or epithelial content. However, this ignores other normal tumour content, such as the vascular cells and muscle, which are recognised separately in the DNN mesenchyme component; this may result in overestimation and explain the higher values for tumour purity compared to DNN epithelial area in this series.

The DNN AI‐based segmentation of tumour compartments is a novel technique, with the practical advantage that it requires only an H&E section, so can generate data cost‐effectively with minimal delay following surgery; this is its first application, to our knowledge, to a series of locally excised rectal cancers. Once the algorithm has been trained the technique is fully reproducible; however, when used on standard H&E sections, as in this clinical setting, the sections may be thicker with a less standardised staining protocol than in the research setting where the algorithm was developed, which could contribute some variability to results. D:I could be considered together with the standard histopathological features in deciding on management following surgery – with high D:I additional treatment, either completion surgery or adjuvant CRT, should be considered or if neither of these is favoured, meticulous surveillance can be instituted. Alternatively, with low D:I, patients can be reassured and follow surveillance with greater confidence. DNN can also be used on biopsy specimens^8^ so could contribute to the initial decision for LE. Further work with larger patient numbers is required to determine whether D:I can also indicate radiosensitivity when considering adjuvant CRT.

The limitations of this study are the relatively small number of patients. We therefore recommend validation of the DNN‐based assessment method in independent cohorts and the prospective clinical trial setting. This is facilitated by rapid adoption of digital pathology for the assessment of primary resection specimens. The algorithm generated here will be made publicly available to facilitate translation. A few of the tumours, considered ‘early’ prior to surgery, were T3. The techniques discussed here require further study in more advanced cancers.

This paper has assessed three measures of stromal content and found some promising prospects for better stratification of locally excised rectal cancers. All techniques were prognostic, emphasising the importance of complex interactions between epithelium, stroma and immune components in determining tumour behaviour. In contrast to colorectal cancer in general,^22^ TSR alone is not so useful in this group. Adding ploidy increases its value, but less than in more advanced cancers. A ratio of ESTIMATE scores is promising, but not immediately useful. AI‐based histomorphology offers a quick and simple addition to standard histopathological assessment with good prognostic value in this patient group. This could provide a valuable extra tool to inform the discussion regarding subsequent management with patients following local excision.

## Conflicts of interest

There are no conflicts of interest.

## Supporting information


**Table S1**. Univariable analysis for disease recurrence
**Figure S1**. Scatter plots showing correlation between DNN measure and gene expression scores in rectal cancers.
