# Supplementary material for: Stromal composition predicts recurrence of early rectal cancer after local excision
Source: Histopathology. 2021 Sep 3;79(6):947–56. doi: 10.1111/his.14438 (PMC8845517; doi:10.1111/his.14438)
Supplement: Supplementary file 1 — Table S1. Univariable analysis for disease recurrence Figure S1. Scatter plots showing correlation between DNN measure and gene expression scores in rectal cancers. [file HIS-79-947-s001.docx]

# Supplemental material

Table S1: Univariable analysis for disease recurrence

|  | No. of patients | No. of events | Hazard ratio (95% CI) | P value (Cox PH model) |
| --- | --- | --- | --- | --- |
| pT pT1  pT2  pT3 | 76  58  9 | 7  16  3 | 1  3.407 (1.40-8.29)  3.983 (1.03-15.5) | 0.00687*  0.04573* |
| Differentiation: well/moderate  Poor | 135  9 | 24  2 | 1  2.03 (0.474-8.68) | 0.341 |
| Lymphovascular invasion: No  Yes | 97  44 | 15  10 | 1  1.47 (0.66-3.27) | 0.347 |
| Positive resection margin: No  Yes | 104  39 | 14  12 | 1  3.02 (1.39-6.55) | 0.00519* |
| DNN D:I | 139 | 26 | 1.01 (1-1.02) | 0.0307* |
| TSR | 143 | 26 | 1.04 (0.997-1.09) | 0.0669 |
| ESTIMATE S:I | 143 | 26 | 1.03 (0.981-1.07) | 0.257 |

* indicates p value <0.05


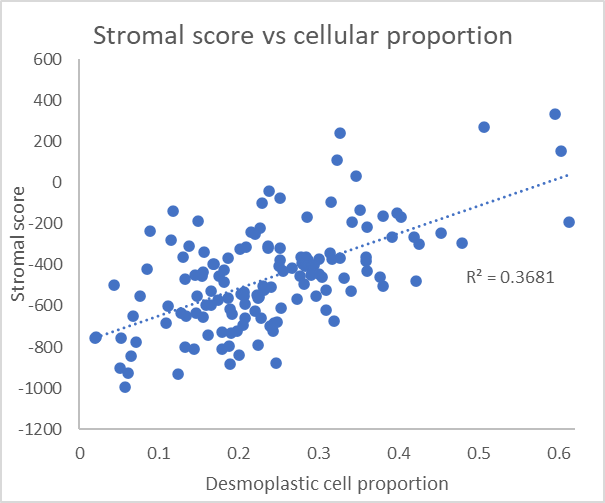


**A**


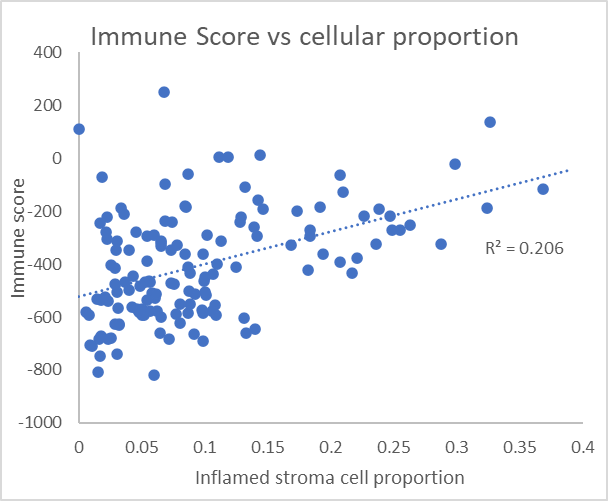


**B**

Figure S2: Scatter plots showing correlation between DNN measure and gene expression scores in rectal cancers. A. Comparison of ESTIMATE stromal score with the cell count proportion of desmoplastic stroma cells determined by DNN in 139 rectal cancers, R^2^ = 0.37. B. Comparison of ESTIMATE immune score with the cell count proportion of inflamed stroma cells determined by DNN in 139 cancers, R^2^ = 0.21.
